# A method of gas sensor drift compensation based on intrinsic characteristics of response curve

**DOI:** 10.1038/s41598-023-39246-8

**Published:** 2023-07-24

**Authors:** Yubing Sun, Yutong Zheng

**Affiliations:** 1 College of Mechanical and Electrical Engineering, Wenzhou University, 325035 Wenzhou, People’s Republic of China; 2 Wenzhou Power Supply Company, Zhejiang Electric Power Corporation, Zhejiang, China

**Keywords:** Engineering, Mathematics and computing

## Abstract

Sensor drift, which is an inevitable and challenging problem in gas sensing, seriously affects the detection performance of sensor. In this study, a new sensor drift compensation method, which is based on intrinsic characteristic of sensory response, is proposed. The dataset of gas sensor for two types of gas with a period of 36 months are collected and two features (one is steady-state feature, another is transient feature) are extracted. Their relationship, which is found to be certain for different months and sensors, is explored. Then, drift compensation method is processed based on this relationship, aiming to make the drifted sensor features adjusted to that of month 1, which is considered as having no drift phenomenon. Moreover, small amount of dataset is necessary for model building and it has strong scalability. Finally, SVM is employed for proving the performance of the drift compensation method proposed in this study. The results show the efficacy of 22 month of continuous monitoring, which has been enough for most application scenario, and almost 20% of increasement of correct classification rate of SVM after drift compensation, which indicates the effect of drift compensation method.

## Introduction

Sensor is a way obtaining information, which is a basic and important tool in information age and has been largely applied in many areas^1,2^. Gas sensor is an indispensable kind of sensor, which is a nondestructive and rapid detection way, having aroused wide concern from researchers due to its close relation to practical applications. Because of those advantages, it has been successfully applied in many areas, such as air quality monitoring^3^, drunk driving^4^, food quality detection^5^, and so on.

Sensor drift is a phenomenon that sensory signal response would gradually and unpredictably change even exposed to the same analyte under identical condition when sensors are operated over a long period of time ^6^. Furthermore, sensor drift is an inevitable problem, which is the characteristic of sensor itself and has plagued the sensor research community for many years. Existing types of gas sensor^7,8^ all belong to chemical sensor, where sensor drift is a serious impairment.

In general, sensor drift can be attributed to two predominant sources ^9,10^: real drift and measurement system drift. The real drift is the main one, which happens due to the chemical and physical interaction processes of the chemical analytes, occurring at the sensing film microstructure. The measurement system drift is produced by the external and uncontrollable alterations of the experimental operating system.

Promoting anti-drift performance of sensor material and proposing a method for drift compensation are two main ways for solving this problem. Developing a new sensor material is cost-consuming and time-consuming. By contrast, proposing a method for drift compensation is relatively easy and has high feasibility. Hence, many studies have been done for drift compensation and two main ways are proposed, which are divided into based on providing reference gas or not.

Drift compensation method based on reference gas is relatively traditional, whose data processing is simple and rapid. Furthermore, acceptable results could be obtained. Ziyatdinov et al.^11^ proposed drift compensation methods based on common principal component analysis combined with reference gas. Good results were obtained. However, practically more devices are needed for supplying extra reference gas, which increases the detection cost and makes the detection process more complicated. Moreover, the choosing of reference gas is another problem. They limit its application scenarios.

For the drift compensation method without reference gas, it is always complex relatively and many studies also have been done. Liu, Chaibou, and Huang^12^ proposed a novel retraining method of multiple self-organizing maps for gas sensor drift compensation. Liu and Tang^13^ developed a novel ensemble method using a dynamic weighted combination of support vector machine (SVM) classifiers. Lei and David^14^ proposed a unified framework called domain adaptation extreme learning machine for drift compensation. Good performances are all obtained. However, those methods are all one step for results obtaining, which contains both drift compensation and gas identification, reducing the flexibility of this method. Furthermore, as the increasement of classified category, the model would be more and more complex. Moreover, large amount of training dataset is necessary for model building and a new model needs to be built when other categories include. Therefore, exploring a new drift compensation method, which needs small amount of training dataset for model building and has strong scalability, is meaningful.

In this paper, a novel drift compensation method based on intrinsic characteristics of response signal, which proceeds without reference gas, is proposed. A parameter, which reflects intrinsic characteristics of gas sensor response signal, is researched. This parameter is invariant for certain sensor and detection time. Furthermore, drift compensation and classification methods are built separately, leading to small amount of training dataset requirement for model building and good model extension ability when other categories include. Moreover, classification algorithm is applied for proving the performance of the new proposed method. The aims of this paper are: (1) to explore the intrinsic characteristics of response signal; (2) to propose a new method realizing drift compensation through intrinsic characteristics; (3) to evaluate the drift compensation performances through support vector machine (SVM)^15^.

## Data acquisition and processing

### Data acquisition

The experiment was carried out during January 2019 to December 2021 (36 months) in a gas delivery platform facility. Eight gas sensors were applied for collecting dataset and two kinds of gases (Ethanol and Ethylene) were tested. The sensors, which were metal-oxide semiconductor (MOS) gas sensors, were bought from Figaro Inc. and the model numbers were TGS 2600, TGS 2602, TGS 2603, TGS 2610-C00, TGS 2611-C00, TGS 2612-D00, TGS2620 and TGS 2630, respectively. For each sensor, the heater module is integrated in sensor for temperature control and 65% RH (relative humidity) is also required for test gas, making the detection environment stability.

In detail, their features display in Table 1 below.

**Table 1:** The features of sensors.

Model number	Features	Target gases
TGS 2600	High sensitivity to total air contaminants	Air pollutants
TGS 2602	High sensitivity to VOCs, ammonia and H2S	Air pollutants
TGS 2603	High sensitivity to amine and sulfur series odor (Trimethylamine, methyl mercaptan, etc.)	Air pollutants
TGS 2610-C00	Quick response to LP gas	Butane Propane
TGS 2611-C00	Quick response to methane	Methane
TGS 2612-D00	Comparable response to %LEL of methane and LP gas	Methane, propane, butane
TGS 2620	High sensitivity to organic solvent vapors	Alcohol, solvent vapors
TGS 2630	High sensitivity to low-flammable refrigerant gases	Refrigerant gases

All the sensors have response to gases Ethanol and Ethylene. The target gases are the ones that corresponding sensor sensitive to. For gas Ethanol, sensor TGS 2600, TGS 2602, TGS 2603, TGS2620 and TGS 2630 have high sensitivity. For gas Ethylene, there is not specific sensor corresponding to. However, the combination of eight sensors gives comprehensive information, making it feasible for Ethylene detection.

The sensors were placed in a 60 ml-volume closed container, which contained inlet and outlet for gas passing through. The flow level of each gas was set as 100 ml/min, ± 1% of accuracy. All the gases were stored in pressurized gas cylinders. Synthetic air was applied as background gas and two tested gases were added to it for all experiment. Moreover, the relative humidity of the tested gas was controlled in 65%. Furthermore, for obtaining stable and effective results, the response of the gas sensor array was measured after seven days preheating period, which is attained via a built-in heater. More details could be seen from the product information at https://www.figarosensor.com/product/sensor/.

For the detection time, 600 s were applied for the gas injection and 500 s for the recovery (cleaning). The sampling rate was set to 60 Hz. The dataset during a period of month 1, 4, 14, 16, 20, 22, 36 was collected and employed for drift compensation research. The data of month 1 was taken as the benchmark, which considered as no drift. The data of month 4 showed the characteristic of the initiating. While, the data of month 14, 16, 20 and 22 showed that of intermediate stage. The data of month 36 showed the characteristic of end stage. The number of samples collected during a period of one month for two gases are showed in Table 2 below.

**Table 2:** The number of samples collected during a period of one month for two gases.

Month ID	Number of samples		
	Ethanol	Ethylene	Total
Month 1	84	88	172
Month 4	82	170	252
Month 14	52	43	95
Month 16	28	40	68
Month 20	264	100	364
Month 22	30	30	60
Month 36	600	600	1200

For model building and testing, the dataset is divided into training set and testing set. 25 samples of each month and gas were selected and applied for model building. Other samples were applied for model testing.

### Data processing

#### Feature extraction

**Figure 1:**
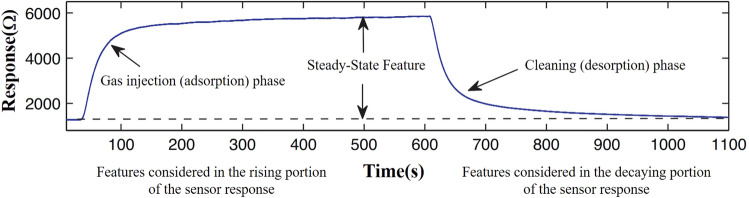
Typical response curve of gas sensor.

Feature extraction ^16^ is an extremely important and inevitable preprocessing step for exploring the data characteristic and applying in real application ^17^. Figure 1 shows a typical response curve of gas sensor, which is also the data collected for analysis.

According to Fig. 1, for gas injection (adsorption) phase, the curve increases first, then become stable. For the cleaning (desorption) phase, the curve decreases first and then returns to the initial stage. Furthermore, the trends of the original curve and drifted curve are similar. On the other hand, the curve trend of desorption phase is largely depended on the characteristic of adsorption phase. The higher stable value means the higher slope values of adsorption and desorption phase. Hence, for reducing the calculation quantity, the data of response curve of adsorption phase are applied for feature extraction.

By contrast, the main difference between them is the stable value and slope of response curve. Hence, four features (one steady-state and three transient features) are selected for drift compensation research in this study. The steady-state feature is defined as the difference of the maximal response value and the baseline.

1 $$ {\text{Fs}} = {\text{Max(}}R) - Min(R) $$

where ${\text{Fs}}$ represents the steady-state feature, ${\text{Max(}}R)$ is maximal response value, $Min(R)$ is the baseline of response curve.

Its normalized version is expressed by the ratio of the maximal response value and the baseline value.

2 $$ \left\| {{\text{Fs}}} \right\| = \frac{Max(R) - Min(R)}{{Min(R)}} $$

where $\left\| {{\text{Fs}}} \right\|$ represents the normalized version of steady-state feature.

The transition feature reflects the dynamics of sensor response and is evaluated based on exponential moving average. The equation presents below.

3 $$ \begin{gathered} {\text{R}}_{{\text{n + 1}}} { = (1 - }\alpha {\text{)R}}_{{\text{n}}} + \alpha {\text{S}}_{{\text{n}}} \hfill \\ {\text{F}}_{{\text{n}}} {\text{ = S}}_{{\text{n + 1}}} {\text{ - S}}_{{\text{n}}} \hfill \\ \end{gathered} $$

where n is the detection time point. ${\text{R}}_{{\text{n}}}$ is response value when detection time point is n. $\alpha$ is the scaling parameter, which amplifies the rising amplitude of adjacent response values, and defined as 0.1, 0.01 and 0.001, respectively. ${\text{S}}_{{\text{n}}}$ could be considered as the parameter containing information of ${\text{R}}_{{\text{n}}}$ and ${\text{R}}_{{\text{n + 1}}} {\text{ - R}}_{{\text{n}}}$. Hence, ${\text{F}}_{{\text{n}}}$ describes the rising amplitude of response value. All the ${\text{F}}_{{\text{n}}}$ are calculated and the average of them is defined as transition feature. Then, three transition features are calculated through three scaling parameters, respectively.

#### Sensor drift compensation method

The process of this method is as follows:

1. Feature extraction and selection: According to the method described above, four features have been extracted, and two of them are selected (one is the steady-state feature, defined as Feature 1 and another is the transient feature, defined as Feature (2) based on the correlation of steady-state feature and transient feature for subsequent analysis.
2. Relationship exploration of the Feature 1 and Feature 2: Build a model of Feature 1 and Feature 2, and use parameters of this model describing their relationship. According to analysis of values of Feature 1 and Feature 2, they basically follow a linear relationship. Hence, the equation is as follow:
  4 $$ {\text{F}}(1)(j)(1) = 1000 \times A(j) \times F(2)(j)(1) $$
  where *i* represents feature number, *j* represents sample number, *k* represents month. Hence, ${\text{F}}(i)(j)(k)$ is defined as the value of feature *i* of sample *j* of Month *k*, ${\text{F}}(1)(j)(1)$ is the value of Feature 1 of sample *j* of Month 1, $F(2)(j)(1)$ is the value of Feature 2 of sample *j* of Month 1, $A(j)$ is the scaling coefficient of sample *j* of Month 1. A is the average of $A(j)$.
3. Relationship exploration of the Feature 1 of Month 1 and other months: Build a model of the Feature 1 of Month 1 and other months, and use parameters of this model describing their relationship. The parameter for all samples is calculated, respectively, and the average of them is defined as $B(k)$, *k* is 4, 14, 16, 20, 22 and 36 representing Month4, Month14, Month16, Month20, Month22, Month36, respectively. The equation is as follows:
  5 $$ B(k) = F(1)(a)(1)/F(1)(a)(k) $$
  where $a$ represents the average value. Hence, $F(1)(a)(1)$ represents the average value of Feature 1 of Month 1, $F(1)(a)(k)$ represents the average value of Feature 1 of month *k*. Therefore, $B(k)$ could be considered as the drift degree of month *k*.
4. Drift compensation: Feature 1 is the feature for drift compensation and applied for next analysis. Parameter $B(k)$ is the chief principal for compensation and parameter A is applied as an auxiliary parameter for improving compensation accuracy. The calculation process is as follows:
  6 $$ Fnew(1)(j)(k) = F(1)(j)(k)/B(k) $$
  where $Fnew(1)(j)(k)$ is the value of Feature 1 of the sample *j* of Month *k* after first step of drift compensation, which should be similar to that of Feature 1 of Month 1. $F(1)(j)(k)$ is the value of Feature 1 of sample *j* of Month *k*.
  7 $$ P(j)(k) = F(1)(j)(k)/[1000 \times F(2)(j)(k)] $$
  where $P(j)(k)$ is the scaling coefficient of sample *j* of Month *k*. Equation (7) is proposed based on Eq. (1). Hence, $P(j)(k)$ should be transformed, making it as close as possible to A for drift compensation. The transformation equation is as follows:
  8 $$ M(j)(k) = P(j)(k) \times A/P(k) $$
  where $P(k)$, which is average value of $P(j)(k)$, could be considered as the actual scaling coefficient of Month *k*. Equation (8) makes the value of $P(j)(k)$ move towards to the value of $A(j)$, which is the scaling coefficient of sample *j* of Month 1. Therefore, $M(j)(k)$ could be considered as the actual scale coefficient of sample *j* of month *k*.
  Real drift and measurement system drift are two predominant sources causing sensor drift. $M(j)(k)$ decreases the real drift. However, the measurement system drift is still existed. Hence, a new parameter is introduced and its equation is as follows:
  9 $$ N(j)(k) = (M(j)(k) + A)/2 $$
  where $N(i)(j)$ is final scale coefficient. In Eq. (9), $M(j)(k)$ and A decrease the real drift and measure system drift respectively, making the compensated result more accurate.
  10 $$ G(j)(k) = Fnew(1)(j)(k)/[1000 \times M(j)(k)] $$
  where $Fnew(1)(j)(k)$ is the value of Feature 1 of the sample *j* of Month *k* after first step of drift compensation. Based on the relationship of Feature 1 and Feature 2 (Eq. (4)), Eq. (10) is obtained. $G(j)(k)$ could be considered as the compensated result of Feature 2 of sample *j* of month *k*.
  11 $$ Ffinal(1)(j)(k) = 1000 \times G(j)(k) \times N(j)(k) $$
  where $Ffinal(1)(j)(k)$ is the final compensated value Feature 1 of the sample *j* of Month *k*. The produce of $Ffinal(1)(j)(k)$ calculation combines Feature 1 and Feature 2, and considers both sensor itself and measure system factors inducing drift, which lead to more accurate results.
5. Drift compensation for all sensors: Apply this method for all sensors and all samples. All the drift compensated features are obtained and applied for subsequence analysis.

According to this method building process, the amount of dataset for model building is small. In this study, 25 samples of each month and gas were selected and applied. Furthermore, the drift compensation and group classification methods are separated leading to strong scalability.

#### Support vector machine

SVM, which is based on the Structural Risk Minimization and Statistic Learning Theory, is considered as one of the most robust and accurate methods used for classification analysis^18^. Furthermore, it has global minimum of the error function and excellent generalization ability of the trained network^19^. It is a linear machine working in the high dimensional feature space formed by the non-linear mapping of the n-dimensional input vector x into a K-dimensional feature space (K > n) with a function. For the results of SVM, samples are considered as points in space and mapped so that the samples of the separated categories are divided by a clear gap that is as wide as possible, which is also the optimal hyperplane to separate two classes.

## Results and discussion

In this part, the sensor drift compensation method proposed is carried out and its performance is discussed according to the results of SVM. Month 1 is set as benchmark, which is considered as no drift. The objective of the drift compensation method is to make the characteristics of features of other months similar to those of month 1.

The data analyzed in this study are: eight steady features and twenty-four transient features in total, which are extracted from eight sensor response curves for representing one sample. Based on Eq. (1)–(3), eight steady-state features are compensated and applied for classification analysis. Twenty-four transient features are compared and eight of them are selected as auxiliary data for drift compensation.

### Sensor drift compensation method

#### Feature extraction and selection

**Figure 2:**
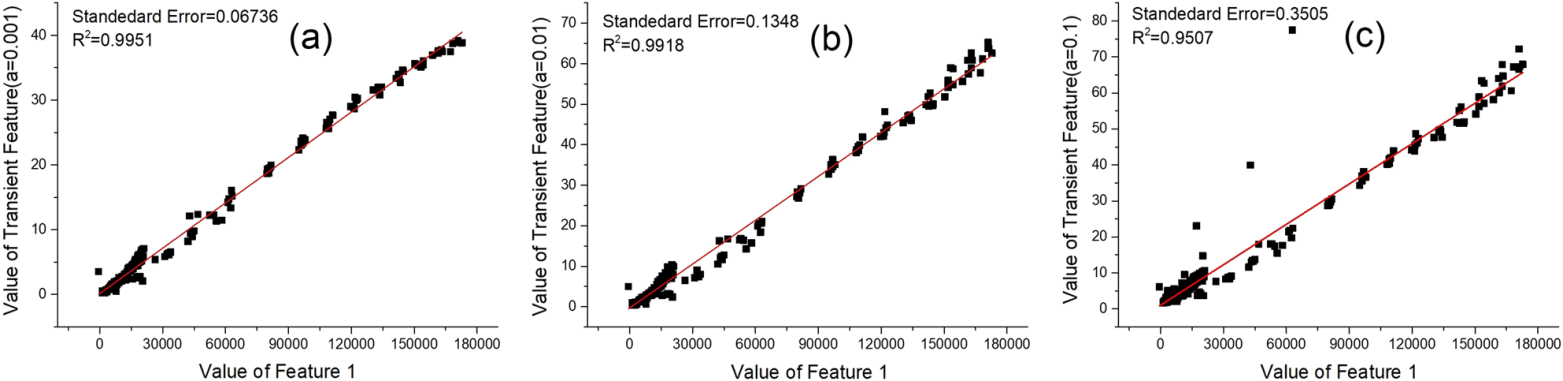
Relevance of Feature 1 and other three transient features. (**a**) $\alpha$ = 0.001, (**b**) $\alpha$ = 0.01, (**c**) $\alpha$ = 0.1

As described above, four features are extracted from one sensor response curve according to Eq. (1) to (3). The steady-state value is defined as Feature 1. Three transient values are compared and selected based on their correlations with Feature 1, and the chosen one is defined as Feature 2. All the samples are employed for feature selection. The scatter plots of Feature 1 and other three features are shown in Fig. 2.

Linear correlation leads to less calculation complex and displays a more obvious visual effect. Hence, it is preferentially applied for selecting appropriate Feature 2. As shown in Fig. 2a, b and c, all plots display strong correlation, which proves the correctness of linear relationship of these two features.

Figure 2a, b and c are the linear relationship of Feature 1 and three transient features with 0.001, 0.01 and 0.1 of scaling parameter, respectively. The fitting correlation coefficients (R^2^) and root mean square error (RMSE) are employed to reflect their fitting performance. According to Fig. 2, Fig. 2a has the best fitting performance, whose standard error is 0.06736, R^2^ is 0.9951. By contrary, Fig. 2c has the worst fitting performance. The reason might be that as the increasement of the scaling parameter $\alpha$, more uncertainty includes, which makes the data fluctuate more greatly. However, the smaller scaling parameter means higher computing cost. For Fig. 2b, its standard error is 0.1348 and R^2^ is 0.9918, which is just a little worse than that of Fig. 2a and also shows good fitting performance. Hence, considering both the fitting performance and calculation complex, the feature with 0.01 of scaling parameter is selected and defined as Feature 2.

#### ***Calculation of A and***$B(k)$

The results of Fig. 2 display the whole relationship of Feature 1 and Feature 2. In this part, their relationships for different months and sensors are discussed in detail. R^2^ and RMSE are employed again and their values are showed in Tables 3 and 4, respectively. The values of RMSE have been normalized, making them comparable.

**Table 3:** The value of R^2^ of different months and sensors.

Month ID	S1	S2	S3	S4	S5	S6	S7	S8
Month 1	0.9836	0.9811	0.9356	0.9435	0.9914	0.991	0.8769	0.8695
Month 4	0.7889	0.4307	0.0363	0.7590	0.6049	0.7219	0.5398	0.4874
Month 14	0.6677	0.6154	0.6828	0.7126	0.3071	0.0239	0.9763	0.8528
Month 16	0.9083	0.992	0.7255	0.7538	0.6742	0.6532	0.4625	0.4743
Month 20	0.9474	0.9862	0.7234	0.7420	0.6006	0.7508	0.5739	0.7648
Month 22	0.8322	0.1798	0.9684	0.9385	0.1864	0.1986	0.5421	0.5775
Month 36	0.9777	0.9363	0.7930	0.8000	0.4411	0.4181	0.7581	0.7476

**Table 4:** The value of RMSE of different months and sensors.

Month ID	S1	S2	S3	S4	S5	S6	S7	S8
Month 1	0.0358	0.0300	0.1127	0.1110	0.0426	0.0368	0.0692	0.0677
Month 4	0.0493	0.0989	0.2468	0.3109	0.2161	0.2245	0.0727	0.0786
Month 14	0.0384	0.0376	0.0317	0.0308	0.1177	0.1150	0.0195	0.0218
Month 16	0.0130	0.0089	0.0671	0.0620	0.0381	0.0417	0.0939	0.0939
Month 20	0.0058	0.0071	0.0266	0.0257	0.0199	0.0269	0.0420	0.0411
Month 22	0.1491	0.1647	0.1479	0.1523	0.1876	0.2078	0.1891	0.1908
Month 36	0.0068	0.0075	0.0111	0.0107	0.0138	0.0138	0.0114	0.0115

The fitting performance of Month 1 is the most important index for the reason of that it is the drift compensation objective. According to Table 3, the values of R^2^ of Month 1 are all higher than 0.86, which indicates good correlation and that parameter A is able to reflect the characteristic of sensor signal response of Month1. Furthermore, values of R^2^ are all acceptable, which proves the correctness of linear correlation of two features again. According to Table 4, the values of RMSE of Month 1 are all less than 0.12, which means a good fitting performance. For all the values in Table 4, most of RMSE are less than 0.15, which also proves the existence of linear correlation of two features.

On the other hand, based on Tables 3 and 4, the fitting performance of Month 1 is the best. For other months, the values of R^2^ and RMSE are fluctuated and most of their fitting performances are worse than that of Month 1. The reason might be environment and measurement system factors, whose uncertainty makes the sensor drift fluctuated.

According to the description above, the relationship of Feature 1 and Feature 2 exists. Furthermore, their relationships for different months and sensors are different, which means that it could be taken as a characteristic for drift compensation. As described in ”Data Acquisition”, 25 samples of each month and gas are selected and applied for parameters A and $B(k)$ calculation.

**Figure 3:**
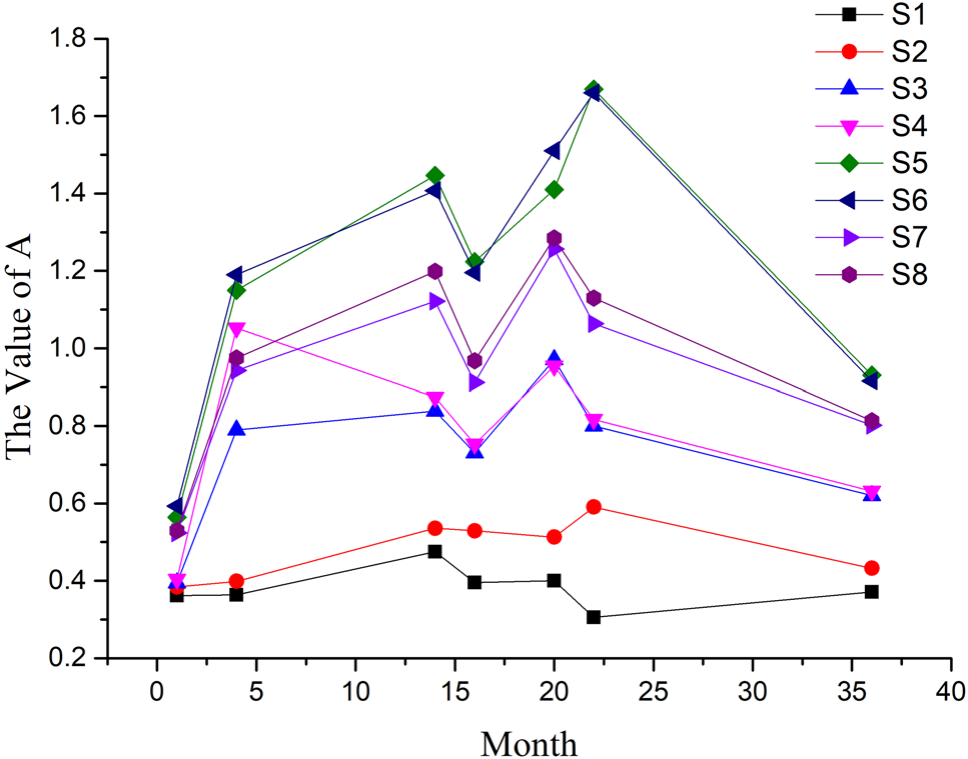
Value trend of A with different sensors and months.

Parameter A reflects the relationship of two features of the same sensor. The values of parameter A with different sensors and months are calculated by Eq. (4) and presented for showing drift phenomena directly and analyzing the drift rule of sensor. Their trends with different months and sensors show in Fig. 3 below.

As shown in Fig. 3, parameter A’s trends of all sensors are similar, which reflects the similarity of drift phenomena. Moreover, the value of parameter A increases first, then fluctuates and finally decreases. For first several months, sensor drift might be influenced by both real drift and measurement system drift, which causes uncertainty and unpredictable. Hence, the values of parameter A fluctuate during this period. Then, drift caused by real drift become dominated with time goes, which has a certain trend and leads to the decrease of the value of parameter A. The results indicate that all sensors have similar drift rule and parameter A is a suitable parameter exhibiting it.

**Figure 4:**
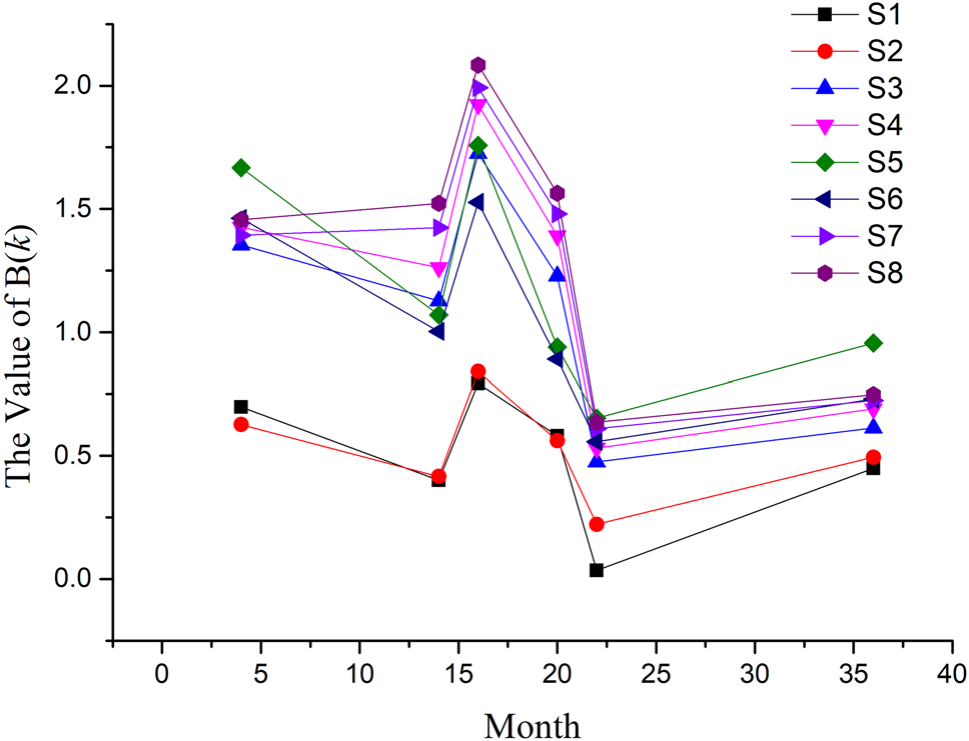
Trend of the value of $B(k)$.

$B(k)$, which reflects the relationship of the Feature 1 of Month 1 and other months, is another important parameter for drift compensation and employed to judge drift degree. The value trends of $B(k)$ with different sensors and months show in Fig. 4 below.

As shown in Fig. 4, the trends of $B(k)$ for all sensors are also similar. It fluctuates first, then become stable. Moreover, the value of $B(k)$ during month 14 to 20 decreases greatly, which increases the drift compensation difficulty. The reason of this trend rule is similar to that of parameter A. The results indicate that all sensors have similar drift pattern, and could be able to compensate using one method.

#### Drift compensation

According to the description above, the relationships between two features of month 1, 4, 14, 16, 20, 22 and 36 are presented. The values of parameters A and $B(k)$ are given. Then, the value of $Ffinal(1)(j)(k)$ is calculated according to the Eqs. (6)–(11) and the drift compensated features for all samples are easily obtained.

In this part, the characteristics of initial and compensated features of sensor 1 detecting gas Ethanol in month 14 present as an example, and their values are compared with that of month 1. Furthermore, their average and standard error are calculated.

The results show that the average of initial feature of month 14 is 5537.32, and its standard error is 2270.74. After drift compensation, its average value becomes 10,599.53, and its standard error is 5740.68. By comparison, the average value of month 1 is 11,149.45, and its standard error is 5102.03, which are similar to those of the dataset after drift compensation. The results prove the effect of the drift compensation method proposed in this study.

Furthermore, the drift compensation features of other months also have similar characteristics, which indicates that the drift compensation method proposed in this study is reliable with high probability.

##### SVM

Feature 1 of all sensors and samples are compensated based on the process above. Then, SVM is applied and the classification performances of original dataset and drift compensated dataset are compared for proving the effect of drift compensation method.

**Figure 5:**
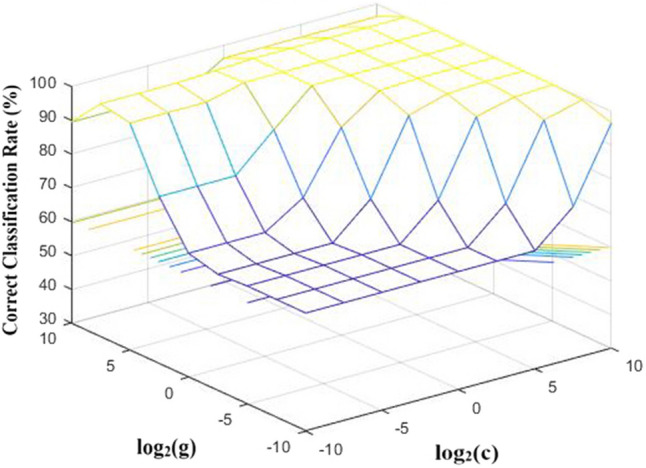
Search of best parameter for the building of SVM model.

For SVM, 180 samples (60 samples for Ethanol, Ethylene and background air, respectively) of Month 1 were selected and applied for building classification model for the reason of that the data of month 1 was the benchmark without drift. The same number of samples of each group helped to balance the importance of each group and improve the classification performance of SVM model. The kernel function was set as Radial Basis Function. For obtaining the optimal SVM model, two parameters, penalty parameter c and kernel parameter g, were optimized through grid search method with the growth of c and g at an interval of 2.5 for obtaining the best c and g. Five-fold cross-validation was applied to estimate the performance of each parameter and the parameters with best cross-validation accuracy were picked. Figure 5 shows the performance of SVM with different combinations of c and g. As shown in Fig. 5, 100% of classification correct rate is obtained when c = 0.1768 and g = 32, which are therefore applied for testing process.

Then, SVM built in previous sentence was applied for classification and the total samples showed in Table 2 were involved. Table 5 presents the results of SVM based on the original dataset and dataset after compensated.

**Table 5:** Results of SVM based on the original data and compensated data.

Month ID	Original data (%)	Compensated data (%)
Month 1	100	100
Month 4	83.73	96.83
Month 14	74.74	88.42
Month 16	66.18	85.29
Month 20	56.04	93.38
Month 22	63.33	85
Month 36	70.67	74.17

As shown in Table 5, the correct classification rates of the dataset of Month 1 are both 100%, which indicates that SVM has good classification performance and proves the effect of SVM. For other months, the original data could be considered as drifted one, whose correct classification rates are all lower than that of Month 1. On the other hand, the results show that the classification performances of compensated data are better than that of the original data for all months, which indicates the effect of this drift compensation method.

In detail, for original data, its correct classification rates decrease as time goes overall. The reason might be that the drift phenomenon become more and more obvious, making SVM model lose efficacy gradually and leading to the decrease of its correct classification rate. For Month 20, its correct classification rate is 56.04%, which has become almost uselessness for the classification of original data. For the compensated data, the correct classification rate obtains more than 20% of increasement compared with that of original data except Month 36 and the average of them reaches to 89.78%, which reflect the good performance of drift compensation method. However, the correct classification rate also decreases as time goes, which indicates that the drift degree could influence the performance of drift compensation method and this influence became bigger with time goes.

As to certain month, the correct classification rates of Month 20 and 22 are low for original data. By contrast, the value of $B(k)$, which is the main parameter for drift compensation, also decreases quickly during this period as shown in Fig. 4. Hence, the reason for bad results might be that the drift degree of original data of Month 20 and 22 has beyond the limit of SVM model. On the other hand, for compensated data, their classification correct rates are both acceptable, which prove that the drift compensation method is still worked in Month 22. For Month 36, the correct classification rate of compensated data is just a little higher than that of original data, which indicates the poor effect and that the drift compensation method does not work well.

According to the discussion above, the drift compensation method proposed in this study is effective when the continuous monitoring time less than 22 month, which has been enough for most application scenario. Hence, the drift compensation method proposed in this study achieves great development of correct classification rate and has practical significance.

## Conclusion

Sensor drift is an inevitable problem in continuous monitoring application. In this study, a new drift compensation method, which is based on the intrinsic characteristics of response signal, is proposed. Small amount of dataset is necessary for model building and it has strong scalability. The dataset of month 1 is taken as no drift and the object of drift compensation. The results show that the characteristic of compensated dataset is similar to that of dataset of month 1, which indicates the efficacy of this method.

Then, SVM is applied for verifying the performance of drift compensation method. The results show almost 20% improvement of correct classification rate, reaching to 89.78%, and the efficacy of 22 month of continuous monitoring, which has been enough for most application scenario.

According to the results above, it is proved that the drift compensation method proposed in this study is effective and this study provides another way for sensor drift compensation.

## Data availibility

The data that support the findings of this study are available from State Grid Corporation of China but restrictions apply to the availability of these data, which were used under license for the current study, and so are not publicly available. Data are however available from the authors upon reasonable request and with permission of State Grid Corporation of China.
